# Early risk stratification and temporal biomarker patterns of trousseau syndrome-related cerebral infarction in lung cancer

**DOI:** 10.3389/fonc.2026.1833717

**Published:** 2026-06-09

**Authors:** Ziyi Xiao, Wenbo Zhang, Yikai Han, Jiaxin Pei, Yushu Deng, Mengmeng Yang, Feng Wang, Taiying Lu

**Affiliations:** The First Affiliated Hospital of Zhengzhou University, Zhengzhou, Henan, China

**Keywords:** Trousseau syndrome, cerebral infarction, lung cancer, risk stratification, temporal heterogeneity

## Abstract

**Background:**

Trousseau syndrome-related cerebral infarction (TSCI) is a severe complication of lung cancer, but tools for early risk stratification remain limited.

**Methods:**

In this retrospective case-control study, we enrolled 94 patients with newly diagnosed lung cancer and TSCI and 94 matched lung cancer controls without stroke at the First Affiliated Hospital of Zhengzhou University between 2021 and 2025. Using cases in which TSCI occurred at diagnosis or within 6 months thereafter, we developed and internally validated a five-variable model based on serum magnesium, international normalized ratio, prothrombin time, albumin-to-globulin ratio, and a history of hypertension. Concurrent-onset (Fulminant) and delayed-onset TSCI subgroups were compared across coagulation, inflammatory, tumor-burden, and nutritional domains. Temporal biomarker patterns were examined using locally estimated scatterplot smoothing (LOESS).

**Results:**

The model showed good discrimination (AUC = 0.861) and sample-specific calibration within the selected case-control sample. Fulminant TSCI showed pronounced abnormalities across coagulation/fibrinolysis, inflammatory, tumor-burden, and nutritional domains. Temporal analyses suggested heterogeneity in biomarker patterns: early-onset TSCI was associated with higher Ki67, neuron-specific enolase, and lactate dehydrogenase, whereas late-onset TSCI was more closely associated with higher triglyceride levels.

**Conclusions:**

These findings identify a set of routinely available clinical variables associated with early high-risk TSCI in patients with lung cancer. The proposed nomogram demonstrated favorable discriminative performance and may provide a useful framework for early risk stratification and clinical surveillance. Further validation in independent prospective cohorts would help to confirm its generalizability and support future clinical application.

## 1 Introduction

Lung cancer remains the leading cause of cancer-related mortality worldwide (1), and the management of its systemic complications has increasingly become a decisive determinant of patient survival and quality of life. According to the GLOBOCAN 2022 database, approximately 2.5 million new lung cancer cases and 1.8 million lung cancer-related deaths are documented globally each year, making it the malignancy with the highest incidence and mortality burden (2). Among its various complications, Trousseau syndrome (TS), defined as malignancy-associated thromboembolic events occurring in the setting of a hypercoagulable state (3), is particularly devastating. When manifesting as acute ischemic stroke (AIS), the condition is referred to as TS-related cerebral infarction (TSCI). Although relatively uncommon, TSCI is associated with substantial disability, recurrence, and early mortality, and remains underrecognized in routine oncology practice (4). TS most commonly complicates highly aggressive malignancies, including lung, pancreatic, breast, and colorectal cancers, a predisposition potentially associated with abnormal tumor-derived mucin secretion (5). Extensive evidence confirms that patients with advanced lung cancer face substantially elevated risks for both venous thromboembolism (VTE) and arterial thromboembolism (ATE) (6–8). These cancer-associated thrombotic events are not only associated with increased mortality but also lead to higher healthcare costs, elevated bleeding risk, and potential delays in cancer treatment. In fact, they represent the second leading cause of death in cancer patients, surpassed only by direct cancer progression (9, 10). Several epidemiological studies have identified TS as a major occult etiology of cryptogenic stroke: approximately 10% of patients with ischemic stroke have a concurrent malignancy (11), and the 1-year incidence of cancer following cryptogenic stroke is approximately 2% (12). Moreover, an estimated 14.6% of cancer patients develop cerebrovascular disease, with approximately half presenting with clinical symptoms (13). Distinct from conventional atherosclerotic or cardioembolic stroke, TSCI is characterized by multifocal ischemic lesions involving multiple vascular territories and a marked propensity for recurrence. Previous studies have reported recurrence rates of cancer-associated thrombosis as high as 34%, with that of ischemic stroke ranging from 15% to 22% (14). The prognosis of this condition is correspondingly poor, with 6-month mortality ranging from 26% to 60% and 1-year mortality exceeding 50% (15, 16). Multiple factors likely contribute to this poor clinical outcome, including poor baseline functional status, persistent hypercoagulability, and the cumulative burden of malignancy-related comorbidities (17). Although advances in targeted therapy and immunotherapy have progressively prolonged survival in lung cancer patients (18–20), the cumulative incidence of TSCI has risen concomitantly. Consequently, this life-threatening complication has become a major bottleneck hindering further improvements in overall survival.

Existing risk stratification tools for cancer-associated thrombosis (CAT), including the Khorana and COMPASS-CAT scores, are primarily calibrated for VTE prediction. Consequently, they exhibit poor performance when applied to arterial thrombotic events such as TSCI (21–23). In contrast, conventional stroke risk scores (e.g., CHA2DS2-VASc) entirely ignore the biological heterogeneity of tumors. As a result, the thrombotic risk of many high-risk lung cancer patients remains unrecognized at cancer diagnosis, leading to the loss of the optimal anticoagulation window. This gap suggests that TSCI risk assessment requires a broader clinical framework than coagulation markers alone.

The emerging concept of immunothrombosis provides a useful framework for understanding this complexity. Immunothrombosis posits that coagulation activation and innate immune responses constitute an inseparable, bidirectional pathological circuit rather than independent processes (24, 25). Within this framework, tumor-derived tissue factor and damage-associated molecular patterns (DAMPs) may trigger extrinsic coagulation cascades and neutrophil extracellular trap (NET) release, while thrombin generation may reciprocally amplify inflammatory signaling. Clinically, this process is likely to involve several interacting dimensions, including coagulation factor consumption, systemic inflammation, nutritional status, and endothelial vulnerability. Serum magnesium is a natural calcium channel antagonist and endothelial stabilizer that may modulate platelet reactivity and NF-kB-driven endothelial inflammation. However, whether magnesium status is independently associated with cancer-associated thrombosis remains incompletely understood. Similarly, the albumin-to-globulin ratio (AGR) simultaneously captures two dimensions: globulin-driven immune activation that promotes NET formation and the erosion of albumin-mediated antithrombotic defenses. This ratio offers a composite readout of the host’s immunothrombotic vulnerability, yet it has been substantially underutilized. A parsimonious model integrating these dimensions may help identify candidate clinical correlates of early high-risk TSCI, but such a model requires cautious interpretation when derived from a retrospective case-control sample.

To address this unmet clinical need, the present study aimed to develop and internally validate a candidate multidimensional model based on routinely available clinical variables within a retrospective matched case-control cohort. The model focused on TSCI occurring concurrently with or within 6 months after lung cancer diagnosis. We also characterized the clinical features of fulminant TSCI and explored whether biomarker patterns differed according to the interval between cancer diagnosis and TSCI onset. These analyses were intended to generate hypotheses for future validation studies rather than to support direct clinical use.

## 2 Methods

### 2.1 Study design and participants

**Figure 1:**
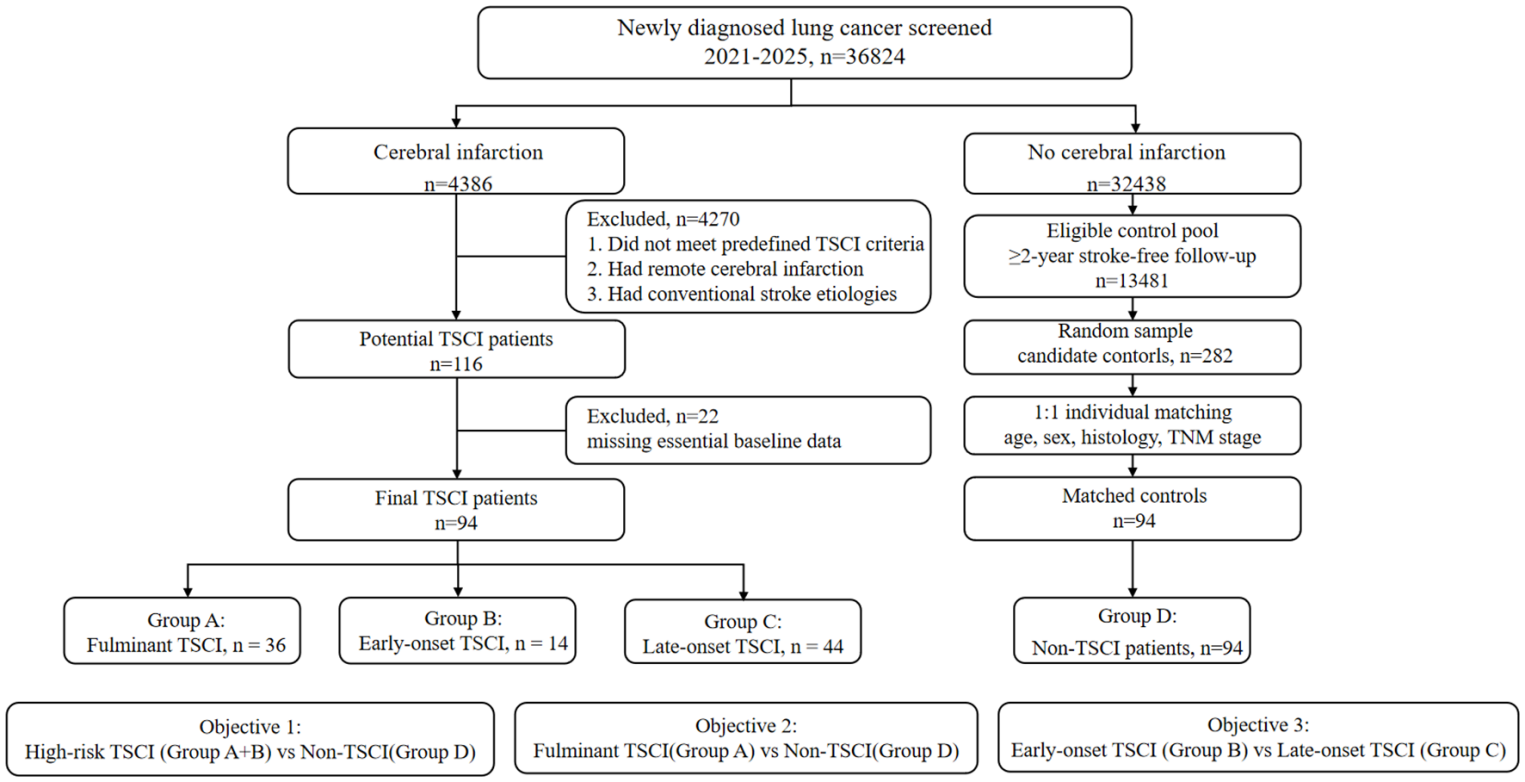
Patient screening, exclusion, control sampling, and study enrollment flowchart.

This retrospective matched case-control study included patients with newly diagnosed lung malignancy treated at the First Affiliated Hospital of Zhengzhou University from January 1, 2021 to December 31, 2025. Clinical data were collected according to prespecified inclusion and exclusion criteria, and the detailed patient screening flowchart is shown in **Figure 1**. During the study period, 36,824 newly diagnosed lung cancer patients were screened according to imaging features, hospitalization records and clinical consultation. Among them, 4,386 had cerebral infarction. Of these patients with cerebral infarction, 4,270 patients were excluded because they did not meet the predefined criteria for TSCI (lacunar cerebral infarction, etc.), had remote cerebral infarction or had established conventional stroke etiologies. Of the remaining 116 potential TSCI patients, 22 were excluded because of missing essential baseline data, leaving 94 TSCI cases for analysis. The TSCI cohort was subdivided by onset timing: Group A (fulminant TSCI, concurrent with cancer diagnosis), Group B (TSCI onset within 6 months after diagnosis), and Group C (TSCI onset more than 6 months after diagnosis).

For the control group, eligible controls were selected from lung cancer patients without cerebral infarction who had at least 2 years of documented stroke-free follow-up. In total, 13,481 patients met these criteria and constituted the eligible control pool. From this pool, 282 candidate controls were randomly sampled, and 94 matched controls were selected by 1:1 individual matching according to age (± 5 years), sex, histological subtype, and TNM stage. Thus, controls were selected by random sampling followed by individual matching rather than consecutive enrollment. Because of the rarity of TSCI, no formal *a priori* sample-size calculation was feasible. Detailed patient baseline data are presented in **Table 1**.

**Table 1:** Baseline demographic, clinical, and laboratory characteristics of the study population.

Variable	Total (n=188)	Group A (n=36)	Group B (n=14)	Group C (n=44)	Group D (n=94)
Time, months	5.50 [0.00, 19.00]	0	5.00 [3.00, 5.00]	21.00 [10.75, 40.75]	NA
Age, year	64.00 [58.00, 70.00]	67.50 [59.50, 73.00]	67.50 [60.50, 73.50]	61.50 [56.50, 70.25]	63.50 [57.00, 68.00]
Sex					
Female	76 (40.4)	12 (33.3)	3 (21.4)	20 (45.5)	41 (43.6)
Male	112 (59.6)	24 (66.7)	11 (78.6)	24 (54.5)	53 (56.4)
Histological type					
Adenocarcinoma	134 (71.3)	26 (72.2)	6 (42.9)	35 (79.5)	67 (71.3)
Squamous cell carcinoma	15 (8.0)	3 (8.3)	2 (14.3)	2 (4.5)	8 (8.5)
Small cell lung cancer /Neuroendocrine carcinoma	27 (14.4)	0 (0.0)	6 (42.9)	6 (13.6)	15 (16.0)
Others	12 (6.4)	7 (19.4)	0 (0.0)	1 (2.3)	4 (4.3)
Smoking history					
No	122 (64.9)	22 (61.1)	8 (57.1)	29 (65.9)	63 (67.0)
Yes	66 (35.1)	14 (38.9)	6 (42.9)	15 (34.1)	31 (33.0)
Drinking history					
No	157 (83.5)	30 (83.3)	11 (78.6)	37 (84.1)	79 (84.0)
Yes	31 (16.5)	6 (16.7)	3 (21.4)	7 (15.9)	15 (16.0)
Diabetes mellitus					
No	167 (89.3)	31 (86.1)	12 (85.7)	40 (90.9)	84 (90.3)
Yes	20 (10.7)	5 (13.9)	2 (14.3)	4 (9.1)	9 (9.7)
Hypertension					
No	126 (67.0)	19 (52.8)	7 (50.0)	31 (70.5)	69 (73.4)
Yes	62 (33.0)	17 (47.2)	7 (50.0)	13 (29.5)	25 (26.6)
Coronary heart disease					
No	171 (91.0)	32 (88.9)	11 (78.6)	42 (95.5)	86 (91.5)
Yes	17 (9.0)	4 (11.1)	3 (21.4)	2 (4.5)	8 (8.5)
Stroke history					
No	179 (95.2)	35 (97.2)	12 (85.7)	42 (95.5)	90 (95.7)
Yes	9 (4.8)	1 (2.8)	2 (14.3)	2 (4.5)	4 (4.3)
TNM stage					
I	2 (1.1)	1 (2.8)	1 (7.1)	0 (0.0)	0 (0.0)
II	9 (4.8)	0 (0.0)	0 (0.0)	3 (6.8)	6 (6.4)
III	47 (25.0)	10 (27.8)	4 (28.6)	10 (22.7)	23 (24.5)
IV	130 (69.1)	25 (69.4)	9 (64.3)	31 (70.5)	65 (69.1)
Height, m	1.68 [1.60, 1.72]	1.68 [1.62, 1.73]	1.70 [1.68, 1.72]	1.65 [1.60, 1.71]	1.67 [1.60, 1.72]
Weight, kg	65.00 [56.00, 72.50]	60.00 [55.00, 70.00]	70.00 [60.00, 80.00]	63.50 [55.75, 75.00]	66.00 [58.25, 71.00]
BMI, kg/m^2^	23.39 [21.40, 25.91]	22.26 [19.82, 23.54]	24.59 [21.33, 27.05]	23.86 [20.49, 27.16]	23.40 [21.84, 25.45]
WBC,10^9^/L	6.73 [5.64, 8.67]	8.94 [7.17, 12.05]	6.41 [6.01, 8.07]	6.40 [5.12, 7.36]	6.62 [5.53, 8.17]
RBC,10^12^/L	4.26 [3.90, 4.61]	4.00 [3.74, 4.48]	4.27 [3.89, 4.50]	4.44 [4.19, 4.59]	4.26 [3.92, 4.71]
Hb, g/L	129.00 [117.00, 140.00]	117.45 [106.50, 131.75]	127.50 [115.00, 138.50]	133.55 [122.00, 141.75]	131.00 [119.28, 142.50]
PLT, 10^9^/L	221.00 [184.75, 269.00]	210.50 [178.00, 288.25]	239.50 [204.75, 262.75]	209.50 [177.75, 270.75]	233.00 [189.75, 266.00]
K, mmol/L	4.13 [3.85, 4.40]	3.98 [3.71, 4.34]	4.06 [3.76, 4.39]	4.17 [3.71, 4.57]	4.16 [3.94, 4.38]
Na, mmol/L	141.00 [139.00, 143.00]	139.00 [136.00, 142.00]	140.55 [138.25, 142.00]	142.00 [139.12, 143.00]	141.00 [140.00, 143.00]
Ca, mmol/L	2.25 [2.17, 2.32]	2.22 [2.12, 2.30]	2.24 [2.19, 2.29]	2.30 [2.17, 2.36]	2.24 [2.17, 2.31]
Mg, mmol/L	0.94 [0.89, 0.99]	0.90 [0.83, 0.94]	0.95 [0.87, 0.96]	0.96 [0.92, 1.01]	0.94 [0.89, 1.00]
Urea, mmol/L	5.08 [4.01, 6.50]	5.07 [3.85, 6.92]	4.90 [4.15, 5.83]	5.10 [3.95, 6.78]	5.17 [4.23, 6.18]
Creatinine, µmol/L	64.00 [57.00, 74.00]	68.00 [63.00, 77.35]	64.00 [54.00, 73.50]	64.00 [56.50, 76.50]	63.00 [56.25, 71.00]
Uric acid, µmol/L	249.00 [206.25, 306.75]	244.00 [195.50, 323.00]	271.00 [225.00, 319.00]	258.00 [200.50, 301.50]	246.00 [212.00, 294.50]
ALT, U/L	14.00 [11.00, 21.00]	14.00 [10.00, 22.00]	23.50 [13.25, 30.75]	15.00 [12.00, 21.50]	14.00 [11.00, 20.00]
AST, U/L	18.00 [15.00, 23.00]	19.50 [14.25, 23.00]	24.00 [18.75, 28.75]	18.00 [15.00, 22.50]	18.00 [14.25, 21.75]
GGT, U/L	23.00 [16.00, 35.00]	28.00 [20.50, 54.00]	30.00 [17.00, 39.25]	21.50 [16.25, 33.50]	20.00 [14.00, 30.75]
ALP, U/L	82.50 [70.00, 103.25]	80.00 [67.75, 123.50]	82.50 [62.50, 96.75]	83.00 [71.00, 99.25]	82.50 [71.25, 98.00]
Total protein, g/L	66.40 [63.00, 70.20]	66.90 [63.20, 69.97]	65.75 [62.88, 69.85]	67.80 [63.20, 72.05]	66.20 [62.83, 69.65]
Albumin, g/L	39.10 [35.90, 42.40]	35.05 [32.45, 39.05]	38.20 [35.50, 41.42]	41.00 [36.70, 44.25]	39.55 [37.60, 42.65]
Globulin, g/L	27.60 [24.30, 29.90]	29.70 [28.12, 32.48]	27.40 [25.75, 29.73]	26.70 [23.75, 28.95]	26.80 [23.33, 29.28]
AGR (ratio)	1.45 [1.27, 1.67]	1.17 [1.02, 1.36]	1.36 [1.28, 1.52]	1.51 [1.36, 1.70]	1.50 [1.34, 1.75]
TBA, µmol/L	4.00 [2.80, 6.15]	2.70 [1.63, 4.18]	3.85 [2.45, 5.23]	4.10 [2.88, 6.00]	4.30 [3.05, 6.55]
CHO, mmol/L	4.18 [3.63, 4.86]	4.26 [3.15, 4.69]	4.50 [3.21, 6.02]	4.33 [3.79, 4.60]	4.14 [3.63, 4.90]
HDL, mmol/L	1.09 [0.93, 1.23]	0.98 [0.88, 1.15]	1.12 [0.99, 1.33]	1.09 [0.94, 1.23]	1.11 [0.96, 1.24]
LDL, mmol/L	2.64 [2.12, 3.04]	2.55 [1.70, 2.96]	2.92 [2.00, 3.62]	2.64 [2.18, 2.96]	2.66 [2.19, 3.04]
GFR, mL/min/1.73m2	94.80 [87.40, 101.34]	90.34 [85.75, 96.29]	93.95 [86.03, 101.42]	94.21 [89.44, 101.39]	97.52 [90.84, 101.63]
LDH, U/L	244.00 [194.25, 312.00]	365.00 [280.50, 462.00]	315.00 [277.00, 354.00]	217.00 [176.50, 258.00]	211.00 [179.00, 278.00]
PT, s	11.20 [10.40, 12.00]	12.40 [11.45, 13.45]	11.35 [11.20, 12.30]	10.80 [10.20, 11.50]	10.95 [10.33, 11.57]
PTA, %	97.70 [89.25, 109.00]	84.00 [74.50, 93.80]	95.00 [84.25, 97.00]	104.00 [93.50, 114.00]	101.65 [93.03, 109.95]
INR	1.01 [0.94, 1.08]	1.12 [1.02, 1.21]	1.04 [1.02, 1.12]	0.97 [0.93, 1.04]	0.98 [0.93, 1.05]
APTT, s	28.60 [26.52, 31.08]	27.20 [26.20, 31.10]	27.70 [25.15, 30.85]	29.10 [27.10, 31.10]	28.60 [26.83, 30.95]
Fibrinogen, g/L	3.42 [2.83, 4.16]	3.68 [2.64, 4.76]	3.60 [3.15, 3.79]	3.40 [3.05, 3.97]	3.34 [2.81, 4.17]
TT, s	15.15 [13.70, 16.98]	15.60 [14.75, 16.80]	15.40 [13.33, 16.60]	14.40 [13.70, 16.60]	15.05 [13.80, 17.10]
DD, mg/L	0.38 [0.16, 1.48]	6.43 [2.30, 12.32]	0.39 [0.22, 1.47]	0.31 [0.14, 0.55]	0.30 [0.11, 0.57]
FDP, µg/mL	2.50 [1.55, 12.92]	32.44 [15.91, 64.27]	2.30 [1.35, 7.77]	1.92 [1.23, 3.16]	2.50 [1.43, 3.81]
CRP, mg/L	4.95 [1.96, 14.72]	33.65 [10.31, 62.55]	4.66 [1.56, 23.98]	4.55 [2.98, 13.72]	3.06 [1.09, 8.08]
PCT, ng/mL	0.07 [0.03, 0.13]	0.10 [0.05, 0.13]	0.06 [0.03, 0.40]	0.07 [0.04, 0.13]	0.05 [0.03, 0.12]
IL6, pg/mL	13.85 [4.11, 38.95]	35.00 [27.27, 55.93]	3.72 [3.54, 40.71]	29.60 [4.24, 53.10]	5.56 [2.94, 10.11]
ESR, mm/h	13.00 [7.00, 20.00]	14.00 [9.50, 20.75]	25.00 [20.00, 28.00]	13.00 [6.00, 15.75]	11.50 [6.80, 18.25]
AFP, ng/mL	2.64 [1.88, 4.13]	3.13 [1.76, 4.66]	2.59 [2.03, 3.30]	2.56 [1.74, 4.41]	2.62 [1.99, 3.90]
CEA, ng/mL	5.81 [2.62, 38.30]	5.82 [2.22, 52.52]	10.18 [2.57, 71.65]	6.42 [2.52, 25.95]	5.50 [2.98, 35.80]
CA125, U/mL	28.20 [13.25, 98.36]	210.00 [128.00, 633.08]	17.98 [10.08, 52.05]	27.60 [13.90, 77.50]	23.68 [11.50, 48.05]
CA199, U/mL	11.68 [5.08, 27.21]	26.92 [5.81, 136.00]	15.76 [5.62, 31.50]	11.18 [5.32, 22.34]	11.47 [4.95, 20.50]
CA153, U/mL	15.80 [9.62, 36.03]	63.80 [39.35, 187.50]	41.50 [12.70, 55.70]	15.80 [11.91, 22.94]	14.05 [9.11, 22.82]
CA724, U/mL	2.48 [1.50, 5.59]	4.19 [1.98, 13.35]	4.67 [1.87, 9.38]	1.65 [1.50, 4.80]	2.26 [1.50, 3.97]
NSCLC, ng/mL	4.77 [2.80, 11.90]	13.60 [7.76, 35.10]	3.25 [2.45, 14.45]	3.68 [2.82, 6.65]	4.21 [2.63, 6.13]
NSE, ng/mL	16.15 [10.43, 25.87]	22.00 [15.50, 28.60]	25.20 [22.80, 33.10]	15.95 [10.62, 27.47]	13.99 [9.52, 18.82]

For TSCI cases, inclusion criteria were as follows:

(1) age >=18 years; (2) lung malignancy confirmed by pathological biopsy; (3) acute ischemic stroke confirmed by cranial MRI; and (4) a diagnosis of TSCI established according to the predefined adjudication criteria described below.

For TSCI cases, exclusion criteria were as follows:

(1) concurrent primary malignancy of another site; (2) stroke attributable to large-artery atherosclerosis, cardioembolism, small-vessel occlusion, or other determined etiology according to TOAST classification; (3) old cerebral infarction without a new acute ischemic lesion; (4) other thromboembolic conditions not considered related to cancer-associated hypercoagulability; and (5) missing essential clinical data. Cases with uncertain classification were reviewed through multidisciplinary discussion involving neurologists and oncologists until consensus was reached.

The diagnosis of Trousseau syndrome was established through multidisciplinary adjudication by experienced neurologists and oncologists according to predefined criteria: (1) active malignancy; (2) acute ischemic stroke confirmed by neuroimaging; (3) exclusion of conventional stroke etiologies according to TOAST classification after routine etiological evaluation; and (4) supportive evidence of cancer-associated hypercoagulability, including elevated D-dimer and/or fibrin degradation products or multifocal infarction patterns. Cases with diagnostic disagreement were resolved by consensus discussion.

Baseline demographic, laboratory, and clinicopathological variables were independently extracted from electronic medical records (EMRs) by two uniformly trained researchers using a standardized data collection form and entered into a structured database. For TSCI cases, laboratory variables, including serum magnesium, were obtained from the first available admission blood tests at the time of TSCI evaluation, before acute stroke-related treatment; for controls, laboratory variables were obtained from the corresponding hospitalization baseline assessment. Data domains included demographic characteristics, laboratory variables, imaging and pathology reports, tumor characteristics, and immunohistochemical data. To assess comparability beyond the matching variables, an additional baseline covariate balance/sensitivity analysis was performed for available non-matched baseline variables, including smoking history, alcohol use, diabetes, hypertension, coronary heart disease, prior stroke history, ECOG performance status, histological grade, age, height, weight, and BMI, with detailed results provided in **Supplementary Table 1**.

### 2.2 Statistical analysis

All statistical analyses were performed using R (version 4.5.2). A two-tailed P-value of <0.05 was considered statistically significant unless otherwise specified. P values for the fulminant subgroup analysis were derived from Wilcoxon rank-sum tests for continuous variables and Fisher’s exact tests for categorical variables.

#### 2.2.1 Development and internal validation of the high-risk TSCI prediction model

To focus on early risk stratification, only TSCI events occurring concurrently with or within 6 months of cancer diagnosis were included in model derivation. Groups A and B (n = 50) therefore comprised the high-risk TSCI group, whereas Group C (late-onset TSCI, >6 months) was excluded from model development because it might represent a biologically distinct subgroup. Group D (n = 94) served as controls, yielding a modeling cohort of 144 patients. Variables with >30% missing data were excluded *a priori*. Remaining missing values were imputed using multiple imputation by chained equations (MICE, predictive mean matching, 5 imputed datasets; pooled via Rubin’s rules). Elastic Net regression (alpha = 0.5; lambda selected by the 1-SE rule) was applied for initial variable selection, followed by AIC-guided model simplification. Model performance was evaluated for discrimination (AUC and DeLong test), sample-specific calibration (calibration curve and Brier score), and exploratory potential net benefit (decision curve analysis). Internal validation was performed with 1,000 bootstrap resamples to estimate optimism and model stability within the selected case-control sample.

#### 2.2.2 Systemic phenotypic quantification of the fulminant TSCI subgroup

To characterize the clinical phenotype of “fulminant” TSCI (Group A, concurrent with cancer diagnosis), non-parametric tests for inter-group comparisons were performed. Systemic deviations across coagulation activation, tumor burden, and immunoinflammatory domains were summarized using median differences, fold changes (FC), and standardized mean differences (SMDs). Effect sizes were categorized as: large (SMD≥0.8), moderate (SMD≥0.5), small (SMD≥0.2), or negligible (SMD < 0.2). Distributional patterns were visualized with stacked bar charts, box plots, and effect forest plots. To account for multiple comparisons, P-values were adjusted using the Benjamini-Hochberg False Discovery Rate (FDR) correction.

#### 2.2.3 LOESS-based non-linear biomarker trajectory modeling

To explore time-related heterogeneity in TSCI, we examined associations between key biomarkers and the interval from lung cancer diagnosis to TSCI onset. Spearman rank correlation was used to assess monotonic relationships, and LOESS smoothing was used to visualize non-linear biomarker trajectories without prespecifying a functional form. Shaded 95% confidence intervals were added to the LOESS curves to illustrate uncertainty in the temporal trends. For descriptive purposes, TSCI was categorized as early onset (<=6 months) or late onset (>6 months).

## 3 Results

### 3.1 Predictive performance and validation of the high-risk TSCI model

**Figure 2:**
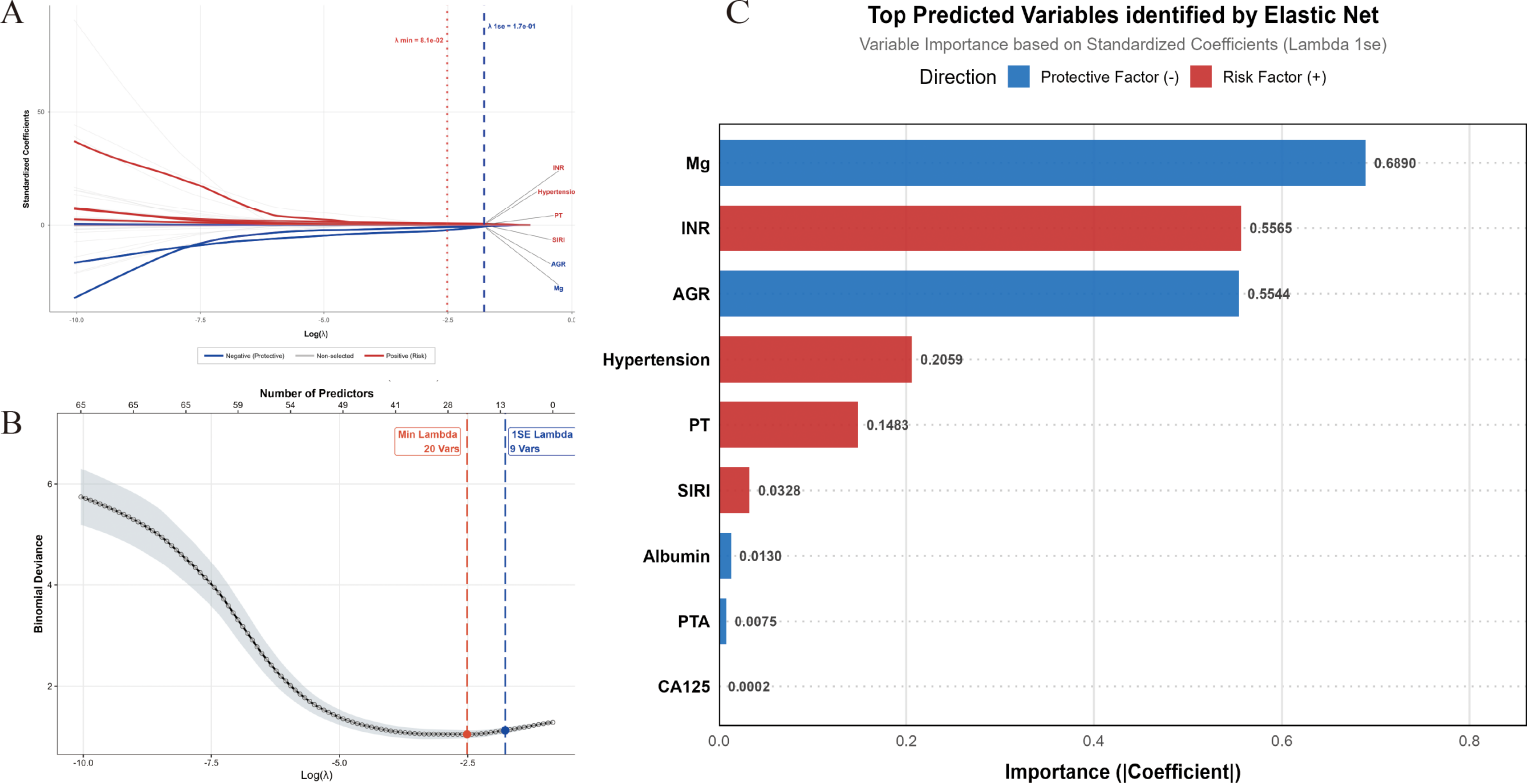
Variable importance ranking derived from 1,000 bootstrap iterations of the elastic net model. **(A)** Coefficient path plot of the elastic net regression, showing standardized coefficients for all variables as a function of log(λ), with the minimum λ and one-standard-error λ marked. **(B)** Cross-validation binomial deviance curve versus log(λ), indicating the minimum and one-standard-error λ values for model selection. **(C)** Horizontal bar chart of variable importance ranking the top predicted variables by absolute standardized coefficients. Blue bars represent protective factors (Mg, AGR, albumin, PTA, and CA125), while red bars represent risk factors (INR, hypertension, PT, and SIRI), with their relative importance coefficients labeled.

Elastic Net regression identified nine preliminary predictive variables: serum magnesium, INR, AGR, PT, PTA, SIRI, CA125, serum albumin, and a history of hypertension. Among these variables, serum magnesium, AGR, serum albumin, and PTA had negative coefficients, whereas INR, PT, SIRI, CA125, and hypertension had positive coefficients. In 1,000 bootstrap resamples, five variables contributed most consistently to prediction (**Figure 2**): serum magnesium, INR, AGR, PT, and a history of hypertension. Serum magnesium had the largest negative coefficient (beta = -0.689; 95% CI, -2.865 to 0), followed by AGR (beta = -0.554; 95% CI, -1.272 to 0), whereas INR had the largest positive coefficient (beta = 0.557; 95% CI, 0 to 1.706). Subsequent AIC-guided stepwise optimization removed four variables (PTA, SIRI, CA125, and serum albumin) whose incremental contributions to model discrimination were minimal and statistically redundant with the retained predictors, yielding a parsimonious five-variable model.

**Figure 3:**
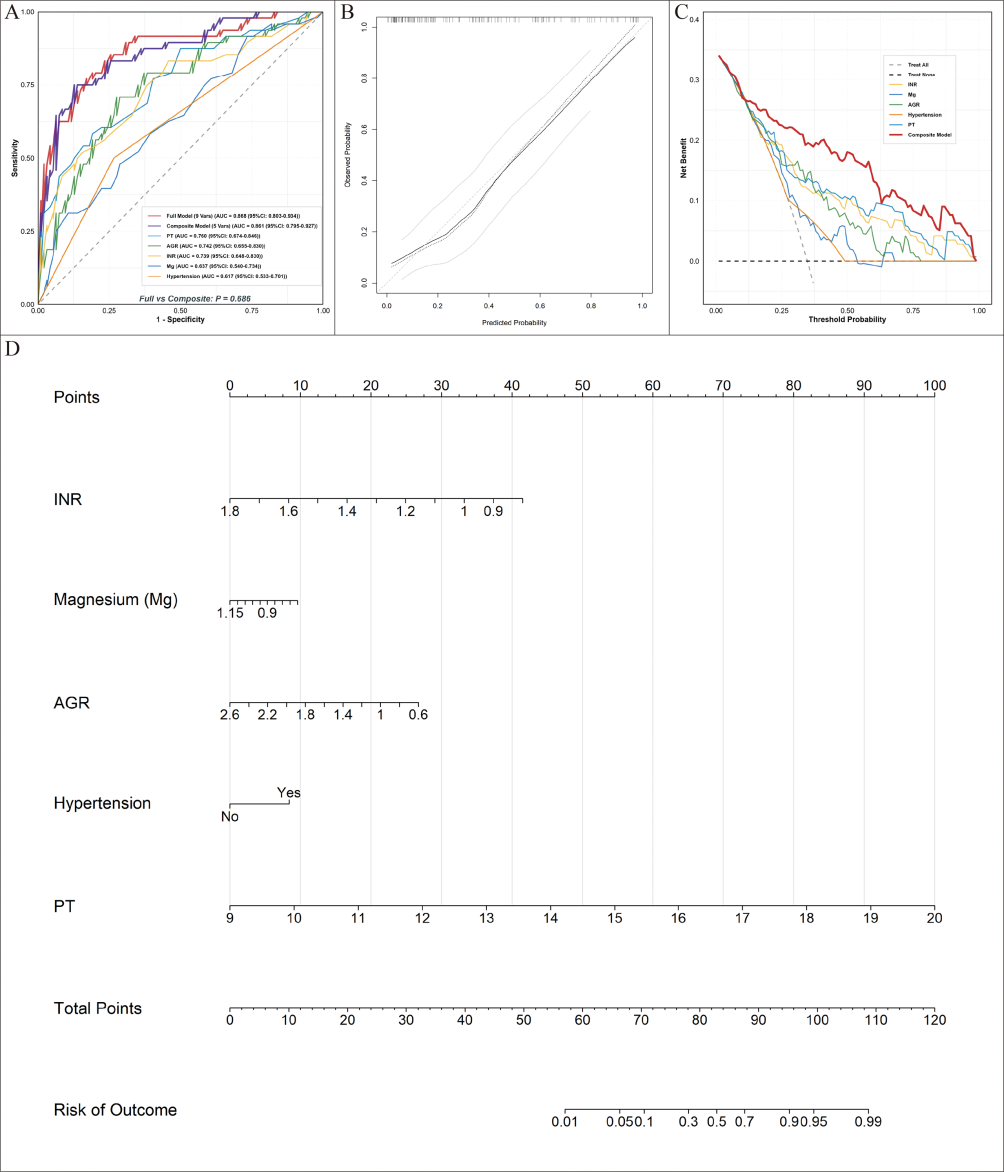
Sample-specific performance evaluation and model-derived estimates of the simplified five-variable model. **(A)** Receiver operating characteristic (ROC) curves showing discrimination within the selected case-control modeling cohort, alongside the bootstrap-corrected area under the curve (AUC). **(B)** Calibration curve assessing sample-specific agreement between model-derived estimates and observed outcomes in the selected case-control cohort, accompanied by the Brier score. **(C)** Decision curve analysis (DCA) showing exploratory, sample-specific estimated net benefit across assumed threshold probabilities. **(D)** Nomogram showing model-derived estimates within the selected case-control cohort based on the identified predictors.

In the selected modeling cohort (n = 144), the 9-variable model yielded an AUC of 0.868. The simplified 5-variable model (INR, serum magnesium, AGR, PT, and a history of hypertension) exhibited strong discrimination within this selected case-control sample, with an AUC of 0.861. It was statistically non-inferior to the full-variable model (DeLong p = 0.686 vs. the full model), while offering greater parsimony and practical feasibility. The simplified model was therefore retained as the preferred parsimonious model within the selected study sample, but this should not be interpreted as evidence that the model is ready for routine clinical implementation. Bootstrap internal validation yielded an optimism-corrected AUC of 0.8423, suggesting limited overfitting within the study sample (**Figure 3A**). Calibration analysis and Brier score were interpreted as sample-specific model-evaluation findings rather than as real-world absolute-risk calibration. The calibration curve closely followed the ideal 45-degree reference line (Brier score = 0.1335; intercept = -0.0538; slope = 0.89) (**Figure 3B**). Decision curve analysis was used as an exploratory, sample-specific assessment of potential net benefit under assumed threshold probabilities within the selected case-control cohort (**Figure 3C**). The corresponding nomogram is presented in **Figure 3D** as a visual representation of model-derived estimates within this sample. The nomogram had a concordance index (C-index) of 0.866 (95% CI: 0.803-0.930).

To further evaluate the sample-specific stability of the model, stratified and sensitivity analyses were performed across subgroups of tumor stage, histological subtype, and sex. Given the limited number of early-stage patients (n = 8), a sensitivity analysis restricted to 136 locally advanced and metastatic cases was performed; the model retained high discriminative performance within this selected sample (AUC = 0.853; 95% CI: 0.783-0.923; DeLong p = 0.784 vs. full cohort; Brier score = 0.1414; MAE = 0.021). Subgroup analyses by histological type yielded AUC values of 0.898 (95% CI: 0.829-0.967) for adenocarcinoma (n = 99) and 0.800 (95% CI: 0.667-0.934) for non-adenocarcinoma (n = 45), with no significant intergroup difference (DeLong p = 0.207) and no significant histology-by-score interaction (p = 0.449). Sex-stratified analyses showed comparable AUC values for male (0.863; 95% CI: 0.785-0.941) and female patients (0.873; 95% CI: 0.752-0.995), with neither a statistically significant difference in AUC (DeLong p = 0.891) nor a sex-by-score interaction (p = 0.518). These findings support internal stability within the selected cohort, but they should not be interpreted as evidence of external generalizability (**Supplementary Figure 1**).

### 3.2 The phenotypic signature of fulminant TSCI (Group A)

Systematic quantitative analysis of fulminant TSCI, defined as TSCI occurring concurrently with lung cancer diagnosis (Group A, n = 36), showed pronounced multisystem abnormalities. Within the coagulation system, D-dimer was 21.4-fold higher than in controls and showed a large effect size (SMD = 0.865, p = 8.55 × 10^-^¹²), accompanied by a significant prolongation of PT (SMD = 1.184, *p* = 2.36 × 10^-8^) and INR (SMD = 1.048, *p* = 8.48 × 10^-7^). Tumor-related and inflammation-related markers were also markedly elevated, including CA125 (SMD = 0.941, *p* = 1.37 × 10^-8^) and SIRI (SMD = 0.959, *p* = 8.25 × 10^-8^). In contrast, AGR (SMD = 1.101, *p* = 6.64 × 10^-7^) and PTA (SMD = 1.182, *p* = 2.65 × 10^-7^) were markedly reduced, consistent with diminished nutritional and coagulation reserve. Detailed fold changes, median differences, and FDR-adjusted P values are provided in **Supplementary Table 2**, and complete SMD quantification data are presented in **Supplementary Table 3**.

**Figure 4:**
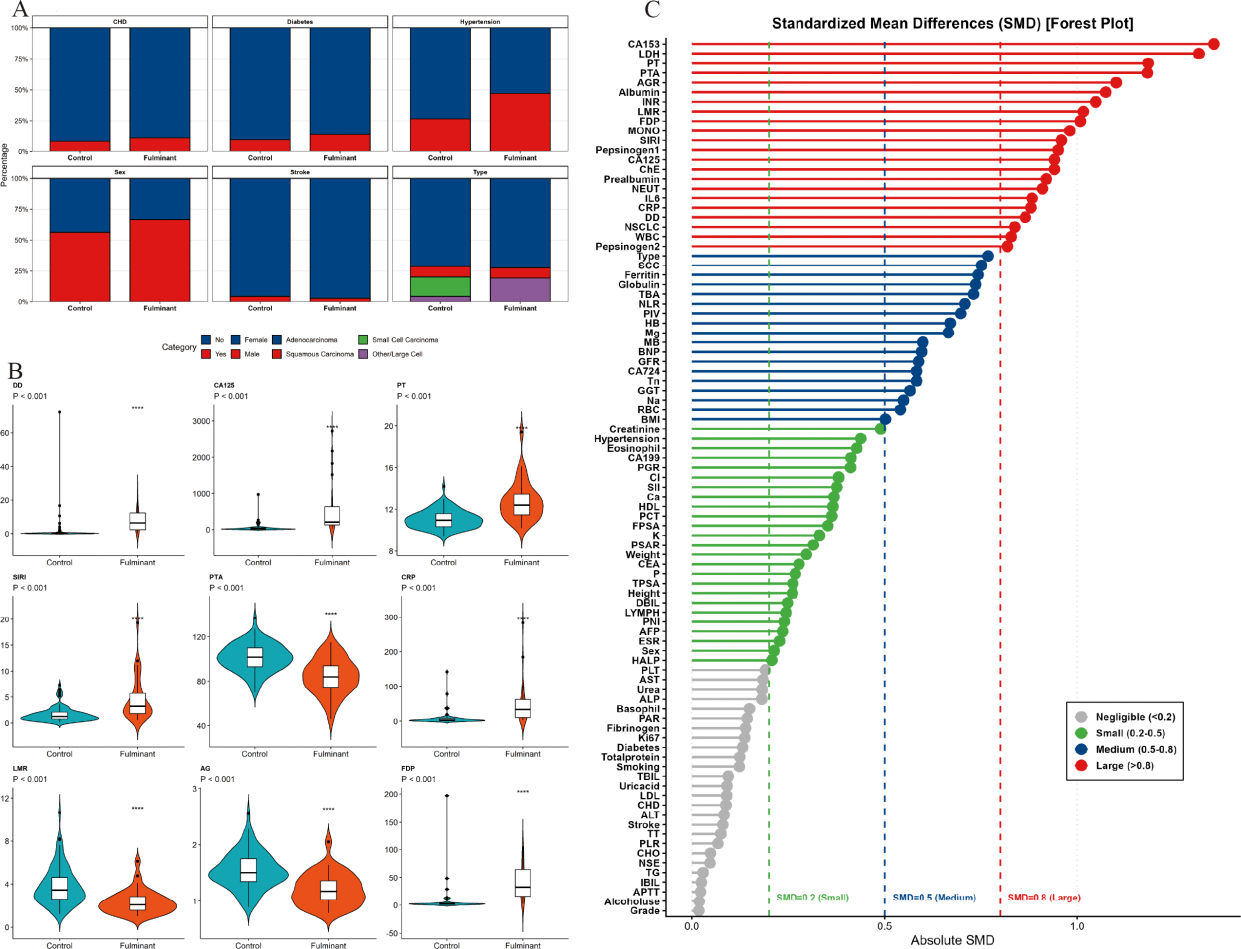
Multidimensional phenotypic characterization of fulminant TSCI. **(A)** Stacked bar charts of categorical-variable distributions. **(B)** Box plots of the nine most discriminating continuous variables. **(C)** Effect forest plot of standardized mean differences (SMDs).

Overall, these findings indicate that fulminant TSCI is associated with marked abnormalities across coagulation/fibrinolysis, tumor-burden, inflammatory, and reserve-related domains. Stacked bar charts identified tumor histology (*p* = 0.003) and a history of hypertension (*p* = 0.035) as the categorical variables that most clearly distinguished fulminant TSCI from controls. Specifically, the fulminant TSCI group had a significantly higher proportion of poorly differentiated tumors and a higher prevalence of hypertension. Smoking status and diabetes mellitus showed directional trends without reaching statistical significance, while sex distribution, stroke history, and coronary artery disease were comparable between the two groups (**Figure 4A**). Box plot analysis of the nine most discriminatory continuous variables revealed a population-level rightward shift in D-dimer, FDP, CRP, and CA125 levels in the fulminant cohort. This distinct distributional difference from the control group was due to genuine group-wide elevation of these indices rather than the influence of outliers. Ultimately, these shifts reflected an order-of-magnitude elevation (D-dimer: 21.4-fold; FDP: 13-fold; CRP: 11-fold) (**Figure 4B**). Forest-plot analyses further showed large effect sizes (SMD > 0.8) for D-dimer, FDP, CA125, and CRP, whereas PCT, HDL, serum calcium, and SII remained in the small-effect range (**Figure 4C**).

### 3.3 Temporal biomarker patterns of TSCI

**Figure 5:**
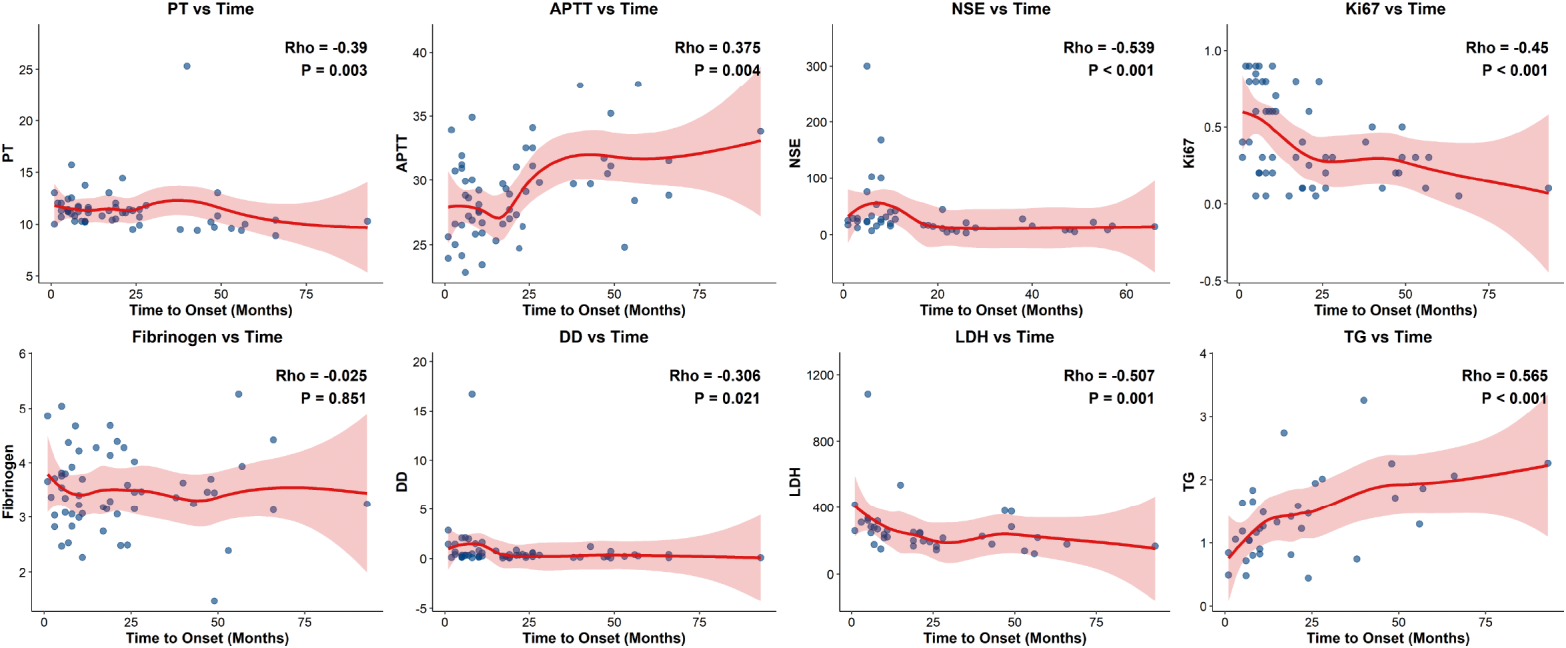
LOESS-fitted non-linear temporal trajectories of key biomarkers with shaded 95% confidence intervals according to onset timing.

To assess whether biomarker profiles varied with the timing of TSCI onset, the TSCI cohort was divided into early-onset (≤6 months) and late-onset (>6 months) subgroups. Tumor-proliferation-related markers were higher in early-onset TSCI: Ki67, NSE, and LDH were each significantly elevated compared with late-onset TSCI (Ki67: ρ = −0.45, *p* = 6.5 × 10^-4^; NSE: ρ = −0.54, *p* = 1.3 × 10^-4^; LDH: ρ = −0.51, *p* = 1.2 × 10^-3^). Full Spearman results are summarized in **Supplementary Table 4**, and detailed values for key variables are reported in **Supplementary Table 5**. LOESS curves showed early peaks in NSE and LDH followed by decline over time; PT and INR displayed a similar pattern of early fluctuation followed by relative stabilization. By contrast, triglyceride levels increased with longer intervals from lung cancer diagnosis to TSCI onset (ρ = 0.56, *p* = 3.3 × 10^-4^), and the corresponding LOESS curve showed a progressive upward trend (**Figure 5**). These findings suggest temporal heterogeneity in the biological correlates of TSCI: earlier events were more closely associated with markers of tumor activity, whereas later events were more closely associated with metabolic disturbance.

## 4 Discussion

In this enriched retrospective matched case-control study, we developed and internally validated a five-variable candidate model for early high-risk TSCI in lung cancer using serum magnesium, INR, PT, AGR, and a history of hypertension. The model showed good discrimination and sample-specific calibration within the selected case-control cohort. In secondary analyses, fulminant TSCI was associated with a particularly severe clinical phenotype, and onset-time analyses suggested that the biological correlates of earlier and later TSCI events may not be identical. These findings identify candidate clinical correlates of early high-risk TSCI while remaining hypothesis-generating and requiring external validation before clinical use.

An important finding of the model was the retention of INR and PT, despite the traditional assumption that thrombosis is primarily associated with shortened clotting times. In cancer-associated thrombosis, however, thrombin generation and coagulation factor consumption may coexist. Mild prolongation of PT/INR may therefore reflect ongoing activation of the coagulation system with depletion of clotting-factor reserve rather than a purely hemorrhagic tendency. This interpretation is consistent with prior reports linking cancer-associated stroke to laboratory features of systemic coagulation activation and consumptive coagulopathy (26–28). The laboratory profile conceptually overlaps with several components incorporated into ISTH-oriented DIC assessment, including marked D-dimer/FDP elevation, PT prolongation, and reduced coagulation reserve. This comparison is intended for pathophysiologic contextualization rather than formal DIC classification. Our data suggest that PT/INR may capture a clinically relevant dimension of TSCI risk not fully represented by single biomarkers such as D-dimer alone.

Serum magnesium also emerged as an inverse correlate of early high-risk TSCI. Although this finding should be interpreted cautiously, it is biologically plausible. Magnesium has been implicated in platelet activation, vascular tone, endothelial function, and inflammatory signaling. Magnesium ions (Mg^2+^) act as endogenous physiological calcium channel antagonists, and hypomagnesemia may facilitate intracellular calcium overload, platelet hyperreactivity, endothelial dysfunction, and NF-kB-related inflammatory activation (29–33). However, the present retrospective data do not support a causal interpretation for magnesium. Hypomagnesemia is common among patients with lung cancer and may reflect cisplatin-associated renal wasting, other systemic treatments, reduced intake, gastrointestinal loss, magnesium supplementation patterns, renal handling, or disease severity. A substantial proportion of fulminant TSCI events occurred before systemic antitumor therapy and anticoagulation, suggesting that platinum-induced magnesium loss cannot fully explain the association in that subgroup. For later-onset cases, however, treatment-cycle timing and cumulative platinum exposure may be relevant confounders. Because these details were incompletely documented, regimen-stratified analysis was not feasible. Magnesium status should therefore be regarded as a candidate marker of thrombotic vulnerability requiring prospective validation, rather than as a proven therapeutic target.

AGR was another independent inverse correlate in the model and may reflect the combined effects of systemic inflammation and nutritional reserve. A low AGR may indicate a host state characterized by a heightened acute-phase response, reduced albumin-mediated buffering and antioxidant capacity, and greater overall physiological vulnerability. Prior work has linked inflammatory activation, neutrophil extracellular traps, endothelial injury, and malnutrition to both cancer-associated thrombosis and ischemic stroke risk. First, globulin elevation driven by IL-6 and TNF-alpha-mediated acute-phase responses can stimulate neutrophils to release NETs. These NETs may perpetuate a pro-inflammatory positive feedback cycle while simultaneously serving as pathological scaffolds that disrupt endothelial integrity and expose tissue factor and procoagulant ligands, ultimately triggering cascading coagulation and thrombosis (34, 35). Second, hypoalbuminemia signals the collapse of the host’s endogenous antioxidant and antithrombotic defenses. Under physiological conditions, albumin not only scavenges reactive oxygen species (ROS) to protect the endothelium, but also exhibits heparin-like antithrombotic activity by binding antithrombin and inhibiting specific coagulation factors (FV, FVII, FXa) (36–38). As these functions deteriorate, ROS-mediated endothelial damage and increased blood viscosity may ensue (39). Large-scale cohort studies have independently linked nutritional deterioration to acute ischemic stroke risk (40). Within that context, AGR may function as a routinely available composite marker. Our findings support its value as a candidate risk-stratification variable within this cohort, while mechanistic interpretation remains provisional.

History of hypertension was retained as an additional predictor. This finding is clinically intuitive because chronic hypertension can contribute to endothelial dysfunction, arterial stiffness, and a prothrombotic vascular environment (41). In the present context, hypertension may represent a vascular substrate on which cancer-related coagulation and inflammatory perturbations exert stronger clinical effects. As with the other predictors, this interpretation is inferential rather than causal.

It is important to emphasize that the apparent model performance should be interpreted in the context of the case-control study design. Because TSCI cases were deliberately enriched in the study cohort, the observed event prevalence does not reflect the true incidence in unselected clinical populations. As a result, model-derived probability estimates do not correspond to absolute clinical risk and may be affected by spectrum bias and prevalence distortion. Consequently, performance metrics such as calibration assessment, Brier score, nomogram-based probability estimation, and decision curve analysis are all influenced by this non-representative sampling framework and should not be interpreted as reflecting real-world predictive accuracy or clinical utility in routine practice. Although bootstrap resampling was used to evaluate internal optimism, this procedure primarily adjusts for overfitting within the study sample and cannot correct for fundamental limitations arising from case-control sampling or discrepancies between study prevalence and real-world disease frequency. Therefore, while the model may provide insight into potential risk stratification patterns within the studied cohort, it should not be interpreted as a validated clinical risk prediction tool for unselected lung cancer populations. The present findings should be regarded as hypothesis-generating and require external validation in prospectively assembled, population-representative cohorts before any clinical application can be considered.

The phenotypic analysis of fulminant TSCI provides complementary clinical context for the model. Patients in this subgroup differed significantly from the matched control group. First, D-dimer (SMD = 0.865) and FDP (SMD = 1.008) levels were elevated by orders of magnitude relative to controls, consistent with extensive systemic coagulation and fibrinolytic activation rather than isolated localized thrombosis alone. This laboratory profile overlaps conceptually with several components of ISTH-oriented DIC assessment, including pronounced D-dimer/FDP elevation, PT prolongation, and reduced coagulation reserve; however, this comparison is intended for pathophysiologic contextualization rather than formal DIC diagnosis. Second, marked elevations of LDH (SMD = 1.315) and CA153 (SMD = 1.354) may reflect high tumor burden or cellular turnover. Third, elevated SIRI (SMD = 0.959) combined with inflammatory globulin elevation suggests systemic immune activation. Correspondingly, reduced AGR, PTA, and serum albumin may indicate diminished nutritional and coagulation reserve. Rather than implying a single discrete syndrome, these findings suggest that fulminant TSCI is associated with simultaneous abnormalities in coagulation/fibrinolysis, tumor burden, systemic inflammation, and physiological reserve. Clinically, this subgroup may lie at the severe end of the TSCI spectrum, although the present data cannot determine whether these abnormalities are causal, consequential, or both.

The temporal analyses further suggest that TSCI is not biologically uniform across the course of lung cancer. Earlier-onset TSCI was associated with higher Ki67, NSE, and LDH, whereas later-onset TSCI was more closely associated with higher triglyceride levels. One possible interpretation is that earlier events are more tightly linked to active tumor biology, whereas later events may occur in the setting of accumulating host metabolic and vascular disturbance. This pattern should be regarded as exploratory. The subgroup sizes were modest, and the LOESS curves were descriptive rather than confirmatory. These temporal patterns may be more relevant to future risk surveillance and monitoring strategies than to immediate differentiation of anticoagulation intensity. Serial monitoring of coagulation biomarkers, particularly D-dimer levels, together with periodic assessment for deep venous thrombosis and repeated neuroimaging when clinically indicated, may be important components of longitudinal management. In this context, lower-extremity venous ultrasonography and brain MRI surveillance could facilitate earlier recognition of subclinical thrombosis or recurrent ischemic events. Although these considerations remain exploratory, they highlight the importance of individualized monitoring approaches in patients with TSCI. Nevertheless, the scientific validity and clinical applicability of these findings still require confirmation in large-scale multicenter prospective studies.

This study has several limitations. First, the model was developed and internally validated within a single-center, enriched retrospective matched case-control sample and has not undergone independent external validation. Its transportability across different populations, clinical settings, and healthcare systems therefore remains uncertain. External validation and recalibration in independent consecutive lung cancer cohorts are required before clinical application. Second, the proportion of TSCI events in the modeling cohort was determined by the case-control design and does not represent the real-world incidence of TSCI among unselected newly diagnosed lung cancer patients. Accordingly, the reported AUC reflects discrimination within the selected modeling sample, and calibration curves, Brier score, nomogram-derived estimates, and decision curve analysis should be interpreted as sample-specific model-evaluation findings rather than as real-world absolute-risk estimates or proven clinical utility. Third, selection bias, information bias, and residual confounding cannot be excluded. Matching was limited to age, sex, histological subtype, and TNM stage, and although additional baseline covariate balance analyses were performed, treatment regimens and anticoagulation exposure could not be fully controlled because they were not uniformly defined pre-baseline variables and were heterogeneously documented in retrospective records. Fourth, serum magnesium may partly reflect treatment-related electrolyte disturbance, renal handling, nutritional status, gastrointestinal loss, magnesium supplementation, or disease severity rather than a direct causal protective mechanism. Fifth, TSCI diagnosis relies partly on excluding other recognized stroke etiologies, and formal inter-rater reliability statistics were not prospectively recorded, leaving some residual risk of misclassification bias. Finally, the mechanistic interpretations proposed here are based on clinical associations rather than direct experimental evidence and require dedicated translational validation.

## 5 Conclusion

In conclusion, we developed and internally validated a five-variable candidate model for early high-risk TSCI in lung cancer using routine clinical variables within an enriched retrospective matched case-control cohort. Serum magnesium, PT/INR, AGR, and history of hypertension were associated with early high-risk TSCI and provided useful discrimination within this selected cohort. Fulminant TSCI was associated with a severe multisystem clinical phenotype, and biomarker analyses suggested time-related heterogeneity between earlier and later events. These findings should be regarded as hypothesis-generating and require external validation, recalibration, and prospective evaluation before clinical use.

## Data Availability

The original contributions presented in the study are included in the article/**Supplementary Material**. Further inquiries can be directed to the corresponding authors.
