# Supplementary figures and images for: Early risk stratification and temporal biomarker patterns of trousseau syndrome-related cerebral infarction in lung cancer
Source: Front Oncol. 2026 Jun 9;16:1833717. doi: 10.3389/fonc.2026.1833717 (PMC13286823; doi:10.3389/fonc.2026.1833717)

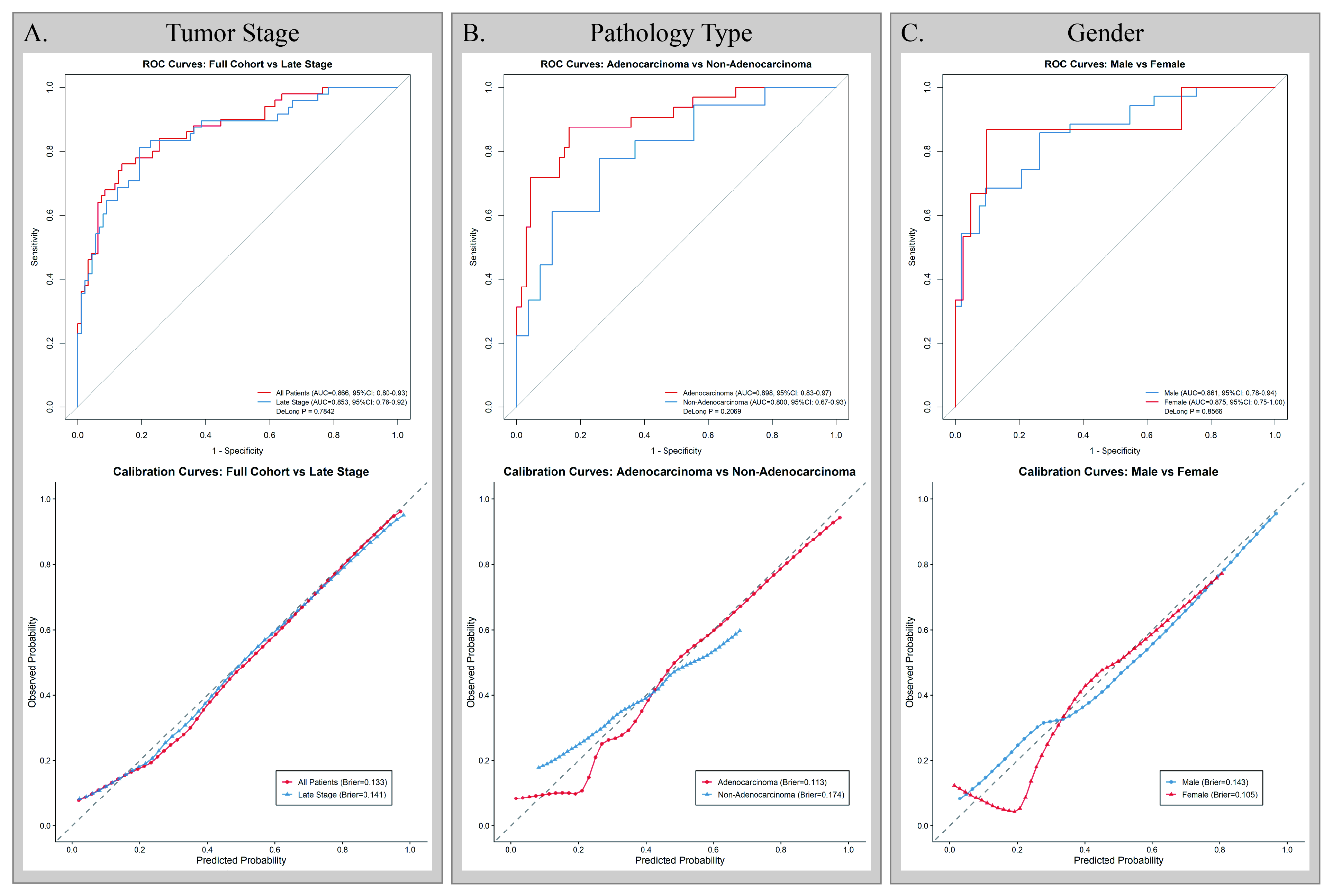

Supplement: Supplementary file 1 [file Image1.tif]
